# Mammalian odorant receptor tuning breadth persists across distinct odorant panels

**DOI:** 10.1371/journal.pone.0185329

**Published:** 2017-09-25

**Authors:** Devin Kepchia, Benjamin Sherman, Rafi Haddad, Charles W. Luetje

**Affiliations:** 1 Department of Molecular and Cellular Pharmacology, University of Miami Miller School of Medicine, Miami, Florida, United States of America; 2 The Leslie & Susan Goldschmied (Gonda) Multidisciplinary Brain Research Center, Bar-Ilan University, Ramat-Gan, Israel; University of Richmond, UNITED STATES

## Abstract

The molecular receptive range (MRR) of a mammalian odorant receptor (OR) is the set of odorant structures that activate the OR, while the distribution of these odorant structures across odor space is the tuning breadth of the OR. Variation in tuning breadth is thought to be an important property of ORs, with the MRRs of these receptors varying from narrowly to broadly tuned. However, defining the tuning breadth of an OR is a technical challenge. For practical reasons, a screening panel that broadly covers odor space must be limited to sparse coverage of the many potential structures in that space. When screened with such a panel, ORs with different odorant specificities, but equal tuning breadths, might appear to have different tuning breadths due to chance. We hypothesized that ORs would maintain their tuning breadths across distinct odorant panels. We constructed a new screening panel that was broadly distributed across an estimated odor space and contained compounds distinct from previous panels. We used this new screening panel to test several murine ORs that were previously characterized as having different tuning breadths. ORs were expressed in *Xenopus laevis* oocytes and assayed by two-electrode voltage clamp electrophysiology. MOR256-17, an OR previously characterized as broadly tuned, responded to nine novel compounds from our new screening panel that were structurally diverse and broadly dispersed across an estimated odor space. MOR256-22, an OR previously characterized as narrowly tuned, responded to a single novel compound that was structurally similar to a previously known ligand for this receptor. MOR174-9, a well-characterized receptor with a narrowly tuned MRR, did not respond to any novel compounds in our new panel. These results support the idea that variation in tuning breadth among these three ORs is not an artifact of the screening protocol, but is an intrinsic property of the receptors.

## Introduction

Mammalian olfaction begins when an odorant molecule reaches the olfactory epithelium within the nasal cavity and binds to an odorant receptor (OR), a G-protein-coupled receptor embedded in the dendritic membrane of an olfactory sensory neuron (OSN) [1]. OR activation initiates a signaling cascade that ultimately depolarizes the membrane and evokes an action potential [2–5]. Each OSN is thought to express a single type of OR [6, 7] and axons of OSNs expressing the same OR converge on two, or occasionally four, glomeruli located in the olfactory bulb [8]. Glomeruli are roughly spherical areas near the surface of the olfactory bulb, made up of incoming OSN axons and the dendrites of mitral and tufted cells that project their axons into the cortex [1]. The mouse genome codes for approximately 1,000–1,200 functional OR genes, while the human genome codes for approximately 350–400 ORs [9–11]. The number of detectable and distinguishable odorants in the environment vastly exceeds these numbers. However, each OR appears to respond to multiple odorants and each odorant appears able to activate multiple ORs, and a combinatorial coding system has been proposed in which the signals of multiple activated ORs are integrated in the CNS to create an odor perception [6, 12].

The compilation of odorant compounds that activate an OR is termed the molecular receptive range (MRR) of that OR [13, 14]. An OR responding only to structurally related compounds can be classified as narrowly tuned, while an OR responding to structurally diverse compounds can be said to be broadly tuned. Tuning breadth is related to, but distinct from MRR, as two broadly tuned ORs could have entirely different MRRs. That is, while both ORs might respond to diverse odorants distributed across a broad expanse of odor space, the specific odorants that activate each receptor could be entirely different. Some of the more extensively studied mammalian ORs appear to have narrowly tuned MRRs: rat and murine I7 receptor is activated by structurally similar aldehydes [13, 15], S6 (MOR42-3) and S50 (MOR42-1) receptors are activated by structurally similar carboxylic acids [16, 17], and M71 (MOR171-2) receptor is activated by benzaldehyde and acetophenone [18]. The presence of much more broadly tuned ORs was, however, suggested by early work with individual amphibian OSNs [19, 20]. Several broadly tuned mammalian ORs have recently been characterized including SR1 (MOR256-3) and Olfr42 (Olfr263, MOR256-31), which are activated by diverse arrays of odorants including linear aliphatic, cyclic, and aromatic structures [12, 21]. Several other mouse and human ORs are activated by diverse structures [22]. Interestingly, a broadly tuned human OR, shown to be responsive to diverse odorant structures, is highly sensitive to a single key food odorant (3-methyl-2,4-nonanedione) and this is the only human OR that responds to this compound [23]. Based on these findings, it has been suggested that this OR may play multiple roles in representing odors [24].

Estimates of OR tuning breadth should not be made by simply counting the number of odorants to which an OR responds. Use of a screening panel containing many odorants with similar structures, or containing odorants restricted to a few sub-regions of odor space, could overestimate or underestimate the tuning breadth of an OR. A better estimate can be achieved through the use of physicochemical metrics to quantitatively assess the distribution of the screening panel and the identified activators across an estimate of odor space [22, 25].

There is also an easily overlooked dilemma regarding the classification of mammalian OR tuning breadths. An odorant panel that achieves broad coverage of odor space must also, for practical reasons, be limited to sparse coverage of the many potential odorant structures. When screened with such a panel, ORs with different odorant specificities, but equal tuning breadths, might appear to have different tuning breadths due to chance. The screening panel might contain many activators of one OR, but few activators of another OR, conveying the false impression that the tuning breadths were different. This concern could be allayed if these ORs maintained their apparent tuning breadths when screened with multiple, distinct odorant panels. However, few ORs have been screened more than once, and when ORs have been characterized in multiple studies there has been substantial overlap among the odorant screening panels [21, 22, 26, 27].

Here, we tested the hypothesis that OR tuning breadth is an intrinsic receptor property, and not an artifact of the screening process, by constructing a new screening panel containing compounds that were broadly distributed across an estimated odor space, but were distinct from previously used compounds. We used this new panel to examine two murine ORs, MOR256-17 and MOR256-22, that we had previously found to be broadly and narrowly tuned, respectively [26]. We also examined MOR174-9, a well-characterized OR with a narrowly tuned MRR [28–30].

## Results

### Distribution of a new odorant panel in an estimated odor space

**Fig 1:**
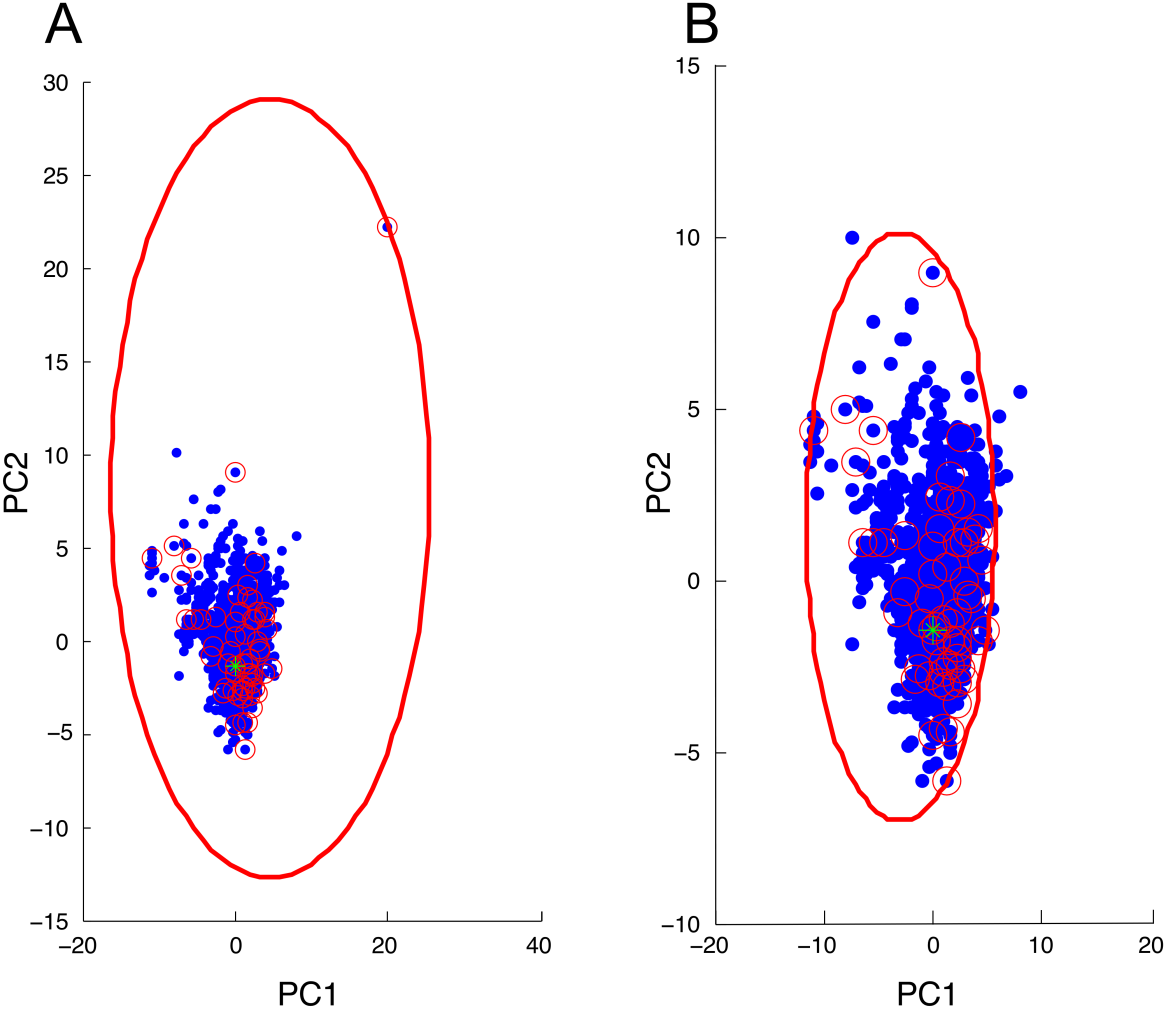
Distribution of our new odorant panel in an estimated odor space. Odor space was estimated using 1595 molecules (blue dots) in a multidimensional space based on 32 physiochemical descriptors and plotted using the first and second principal components. The 60 odorants in our screening panel are indicated with small red circles. One odorant (*trans*-cinnamaldehyde, green star) was included from our previous panel for reference. (A) The entire odor space is shown, including an extreme outlier (iodoform). The smallest hypersphere (indicated by the large red ellipse) that could encompass all odorants in our screening panel (including iodoform) had a radius of 43.0. (B) A close-up view of the region containing all of the molecules (excluding iodoform) is shown. The smallest hypersphere (indicated by the large red ellipse) that could encompass the odorants in our screening panel (excluding iodoform) had a radius of 15.5.

In Fig 1, we estimated odor space using 1595 molecules common to olfactory studies (indicated by blue dots) in a multidimensional space based on 32 physiochemical descriptors [25] and plotted this odor space using the first and second principal components (S1 Table). To assemble compounds for a new screening panel, we used a previously published set of odorant selection groups [25] to choose compounds that adequately sampled various regions of the estimated odor space. We selected a panel of 60 odorants (indicated by red circles in Fig 1). Names and PubChem CID numbers for these compounds are provided in Table 1. One compound (*trans*-cinnamaldehyde, indicated by a green star in Fig 1) was included from the previous panel, because this compound robustly activates both MOR256-17 and MOR256-22 [26]. The other 59 compounds in the new panel were chosen to be distinct from the compounds in our previous screening panel [26]. The extreme outlier in Fig 1A is iodoform. While it may appear that non-sampled odor space exists between iodoform and the other compounds, it is important to understand that odor space is not continuous. This empty area represents combinations of molecular descriptors that do not code for known odorants.

**Table 1:** Composition of odorant mixtures (with CID numbers).

**Mixture 1**	**Mixture 4**
α-hexylcinnamaldehyde (1550884)	octanoic acid (379)
bromobenzene (7961)	2,3-butanedione (650)
methyl hexanoate (7824)	cycloundecanone (13420)
isoamyl phenylacetate (7600)	(-)-terpinen-4-ol (5325830)
3-octanol (11527)	α,α-dimethylbenzenepropanol (7632)
2-isobutyl-3-methoxypyrazine (32594)	*trans*-cinnamaldehyde (637511)
ethyl decanoate (8048)	amyl methyl sulfide (15620)
L-menthyl acetate (220674)	piperonal (8438)
dimethyl succinate (7820)	6-bromohexanoic acid (20210)
thiazole (9256)	pyrazine (9261)
**Mixture 2**	**Mixture 5**
cyclohexane (8078)	1,4-diaminobutane (1045)
(R)-(+)-β-citronellol (101977)	4-hydroxybenzaldehyde (126)
suberoyl chloride (534653)	2,2'-thiodiacetic acid (31277)
ethyl benzoate (7165)	pimelic acid (385)
toluene (1140)	5-bromovaleric acid (16368)
methyl eugenol (7127)	hydantoin-5-acetic acid (95492)
thiophene (8030)	iodoform (6374)
γ-decalactone (12813)	isoborneol (6321405)
4-methylquinoline (10285)	6-acetyl-1,1,2,4,4,7-hexamethyl tetralin (89440)
isophorone (6544)	2,5-dimethyl-2,5-dihydroxy-1,4-dithiane (62105)
**Mixture 3**	**Mixture 6**
cyclopentanone (8452)	1,4-dimethoxybenzene (9016)
2-methoxypyrazine (18467)	naphthalene (931)
geranyl butyrate (5355856)	musk ketone (6669)
octane (356)	linoleic acid (5280450)
(-)-trans caryophyllene (5281515)	pyridine (1049)
methyl cedryl ether (88288)	trimethylamine (1146)
methyl isonicotinate (227085)	isovaleric acid (10430)
(1s)-(+)-3-carene (443156)	2,5-dihydro-2,4,5-trimethylthiazoline (263626)
4-methyl-5-thiazoleethanol (1136)	5-ethyl-3-hydroxy-4-methyl-2(5H)-furanone (61199)
1,3,4,6,7,8-hexahydro-4,6,6,7,8,8-hexamethylcyclopenta-	
-[g]-2-benzopyran solution (91497)	**Additional Compounds**
	cyclodecanone (73918)
	2-heptanone (8051)
	eugenol (3314)

### MOR256-17 responds to a large set of compounds with diverse chemical structures

We chose MOR256-17 as an example of an MOR thought to be broadly tuned because a previous detailed characterization of this receptor by our group showed responsiveness to a wide variety of odorant structures [26] and use of this receptor in the current study would then allow characterization under highly comparable experimental conditions. We expressed MOR256-17 in *X*. *laevis* oocytes, along with human Gα_olf_ and human CFTR, to allow for an electrophysiological assay of receptor function [16]. The receptor was screened with our newly constructed odorant panel. Odorants were applied in 6 mixtures (Table 1) containing 9 or 10 compounds, each present at a concentration of 30 μM. Compounds were distributed among the 6 mixtures so as to have a diverse set of structures in each mixture. We chose to use a screening concentration of 30 μM for two reasons. First, odorants activating many previously characterized mammalian ORs have EC_50_ values in the low to mid-μM range [29, 31]. Second, the previous characterization of the MRR of MOR256-17 [26], to which we planned to compare the results of the current study, used a 30 μM screening concentration. 2-heptanone, a potent agonist of this receptor [26], was used to normalize the responses of individual oocytes.

**Fig 2:**
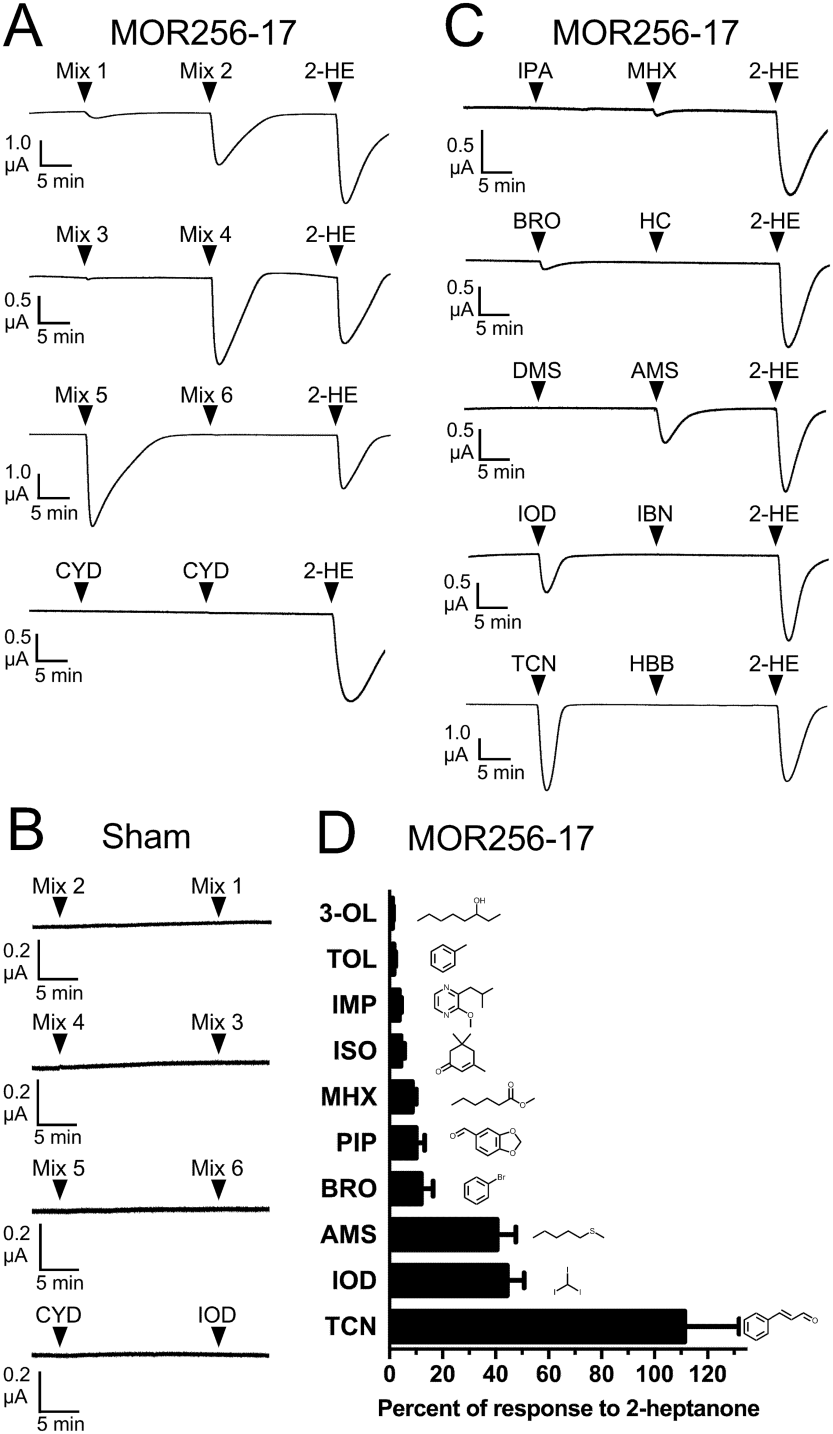
MOR256-17 remains broadly tuned, responding to diverse chemical structures. (A) Current recordings of oocytes expressing MOR256-17, Gα_olf_ and CFTR. Odorants were screened in 6 mixtures, with each odorant present at 30 μM. Cyclodecanone (CYD) was screened individually. An application of 2-heptanone (2-HE) is included at the end of each trace for normalization. Representative traces are shown (n = 5–8). (B) Representative current recordings (n = 3–6) of the six odorant mixtures, as well as iodoform (IOD) and cyclodecanone (CYD), applied to sham (water) injected oocytes. Each odorant was present at 30 μM. (C) Representative current recordings of oocytes expressing MOR256-17, Gα_olf_ and CFTR responding to individual odorants applied at 30 μM. Four of the nine novel odorants we identified as activators of MOR256-17 are shown: methyl hexanoate (MHX), bromobenzene (BRO), amyl methyl sulfide (AMS) and iodoform (IOD). Also shown is the previously identified MOR256-17 ligand, *trans*-cinnamaldehyde (TCN), that was included in Mixture 4. Several inactive compounds are also shown: isoamyl phenylacetate (IPA), α-hexyl cinnamaldehyde (HC), dimethyl succinate (DMS), isoborneol (IBN), and 1,3,4,6,7,8-hexahydro-4,6,6,7,8,8-hexamethylcyclopenta-[g]-2-benzopyran (HBB). An application of 2-HE is included at the end of each trace for normalization. (D) Responses to nine newly identified odorant ligands were normalized to the response to 2-HE and are presented as mean ± SEM (n = 6–9). In addition to the compounds shown in panel C: 3-octanol (3-OL), toluene (TOL), 2-isobutyl-3-methoxypyrazine (IMP), isophorone (ISO) and piperonal (PIP).

MOR256-17 responded to mixtures 1, 2, 4 and 5 (Fig 2A). Sham (water) injected oocytes did not respond to any of the mixtures (Fig 2B). Individual odorants from each of the active mixtures were then screened against the receptor. MOR256-17 responded well to the positive control *trans*-cinnamaldehyde. Nine novel activators were discovered, as MOR256-17 responded moderately to iodoform, amyl methyl sulfide, bromobenzene, piperonal, and methyl hexanoate; and responded modestly to isophorone, 2-isobutyl-3-methoxypyrazine, toluene, and 3-octanol (Fig 2C and 2D, S2 Table). Thus, MOR256-17 responded to a diverse group of odorants, including linear aliphatic as well as cyclic (aliphatic and aromatic) structures. These odorants possessed a variety of functional groups, including aldehyde, alcohol, ether, ester, ketone, sulfide, and halogen.

Mixture 5 elicited receptor responses that exceeded that of the normalizer (Fig 2A). However, the only active odorant in this mixture was iodoform, which yielded responses that were 44 ± 6% of the normalizer response. While most of the odorants are unlikely to react with each other, the unique chemical properties of iodoform suggest reactive potential. To investigate this possibility, iodoform was mixed with each of the other nine odorants in mixture 5 and these binary mixtures were then tested (30 μM of each compound). The binary mixtures evoked highly variable individual responses ranging from 9 ± 2% to 273 ± 108% of the normalizer response (S2 Table). This suggested that iodoform was indeed reacting with other components of the mixture to yield novel structures, some of which were highly active. These results further underscore the broad tuning of MOR256-17. While the identity of the new odorants was not pursued, iodoform was removed from mixture 5 due to this reactivity and was subsequently tested individually.

### MOR256-22 responds to compounds with similar chemical structures

**Fig 3:**
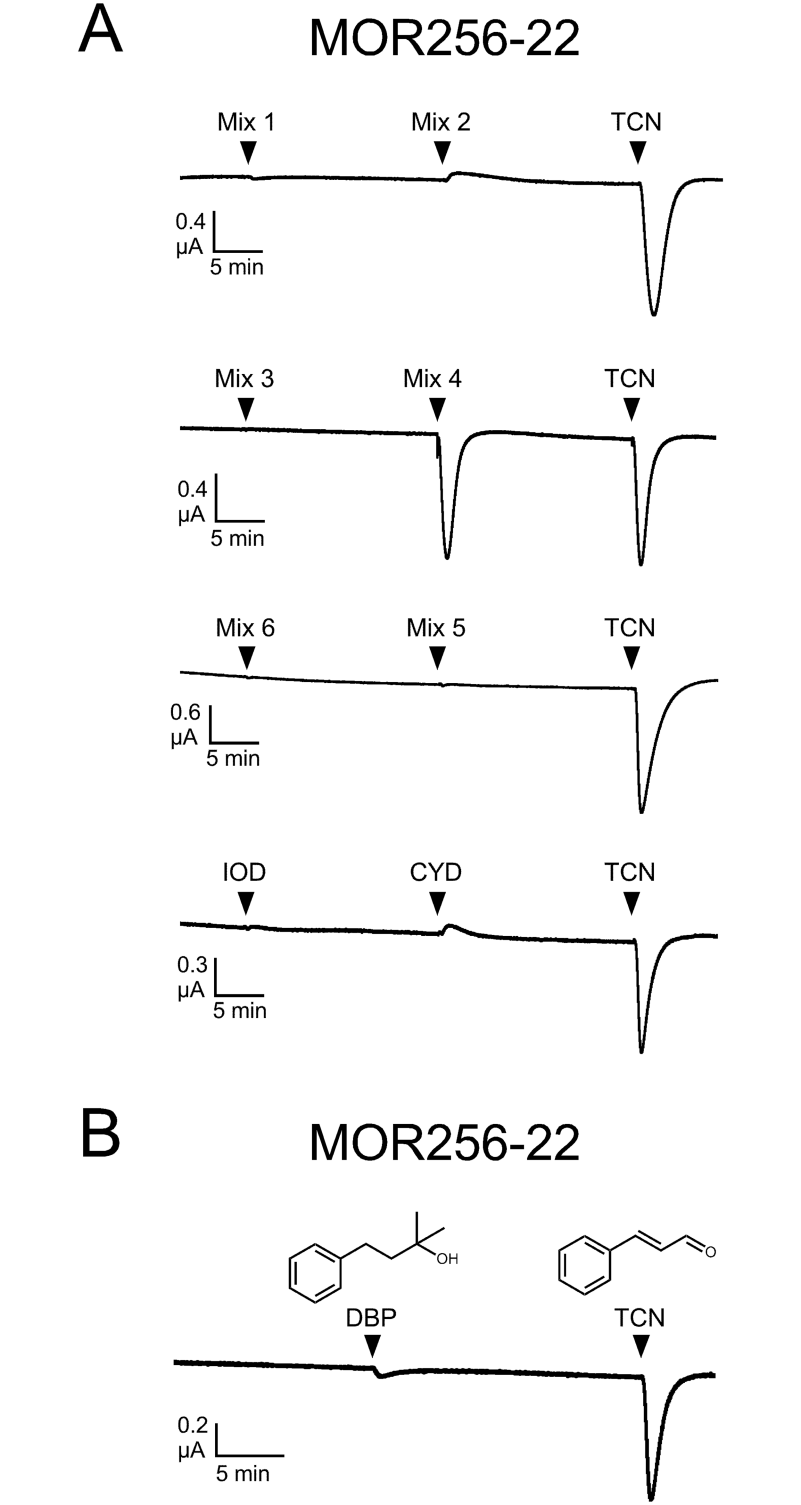
MOR256-22 remains narrowly tuned, responding to structurally similar odorants. (A) Current recordings of oocytes expressing MOR256-22, Gα_olf_ and CFTR. Odorants were screened in 6 mixtures, with each odorant present at 30 μM. Cyclodecanone (CYD) and iodoform (IOD) were screened individually. Responses were normalized to the *trans*-cinnamaldehyde (TCN) response. Representative traces are shown (n = 4–8). (B) Current recording of an oocyte expressing MOR256-22, Gα_olf_ and CFTR responding to individual odorants. MOR256-22 only responded to the previously identified odorant ligand (*trans*-cinnamaldehyde) and α,α-dimethylbenzenepropanol (DBP), a structurally similar novel activator. A representative trace is shown (n = 9).

Next, we chose MOR256-22 as an example of an MOR thought to be narrowly tuned because a previous detailed characterization of this receptor by our group showed responsiveness to a limited set of odorant structures [26]. We screened MOR256-22 with the new odorant panel. *Trans*-cinnamaldehyde, the positive control included in mixture 4, was used to normalize responses of individual oocytes. Only mixture 4 evoked a response from MOR256-22 (Fig 3A). In addition to *trans*-cinnamaldehyde itself, we identified one novel odorant activator of MOR256-22 within mixture 4. α,α-dimethylbenzenepropanol elicited responses that were 6 ± 2% of the normalizer response (Fig 3B, S3 Table). *Trans*-cinnamaldehyde and α,α-dimethylbenzenepropanol have similar structures: both possess a propyl group attached to a benzene ring, with a terminal decoration containing an oxygen.

### MOR174-9 does not respond to the panel of odorants

**Fig 4:**
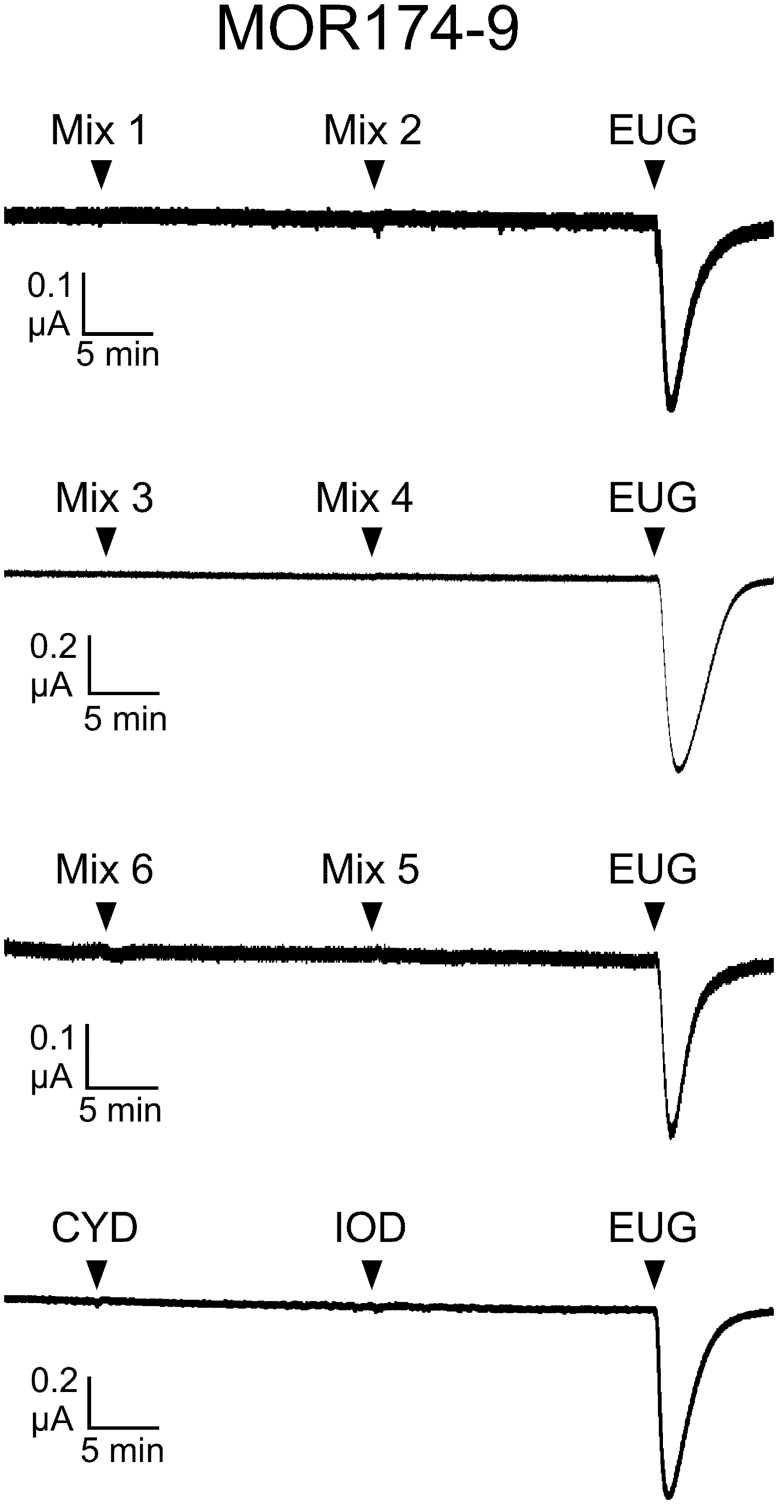
MOR174-9 does not respond to the new panel of odorants. Current recordings of oocytes expressing MOR174-9, Gα_olf_ and CFTR. Odorants were screened in 6 mixtures, with each odorant present at 30 μM. Cyclodecanone (CYD) and iodoform (IOD) were screened individually. Responses were normalized to the eugenol (EUG) response. Representative traces are shown (n = 4–5).

We chose MOR174-9 (mOR-EG) as another example of an MOR thought to be narrowly tuned because we and others have found that this MOR responded to a limited set of odorant structures [26, 28–30]. None of the odorants in our new screening panel evoked responses from MOR174-9 (Fig 4). Because MOR174-9 responds well to eugenol, this receptor might be expected to display activity during application of mixture 2, which contained methyl eugenol (at 30 μM), but no activity was observed. However, application of a higher concentration of methyl eugenol (300 μM) could elicit a modest response from this receptor (25 ± 8% of normalizer response, n = 5, S4 Table), consistent with a previous report [32].

### The molecular receptive range remains broad for MOR256-17 and narrow for MOR256-22

**Fig 5:**
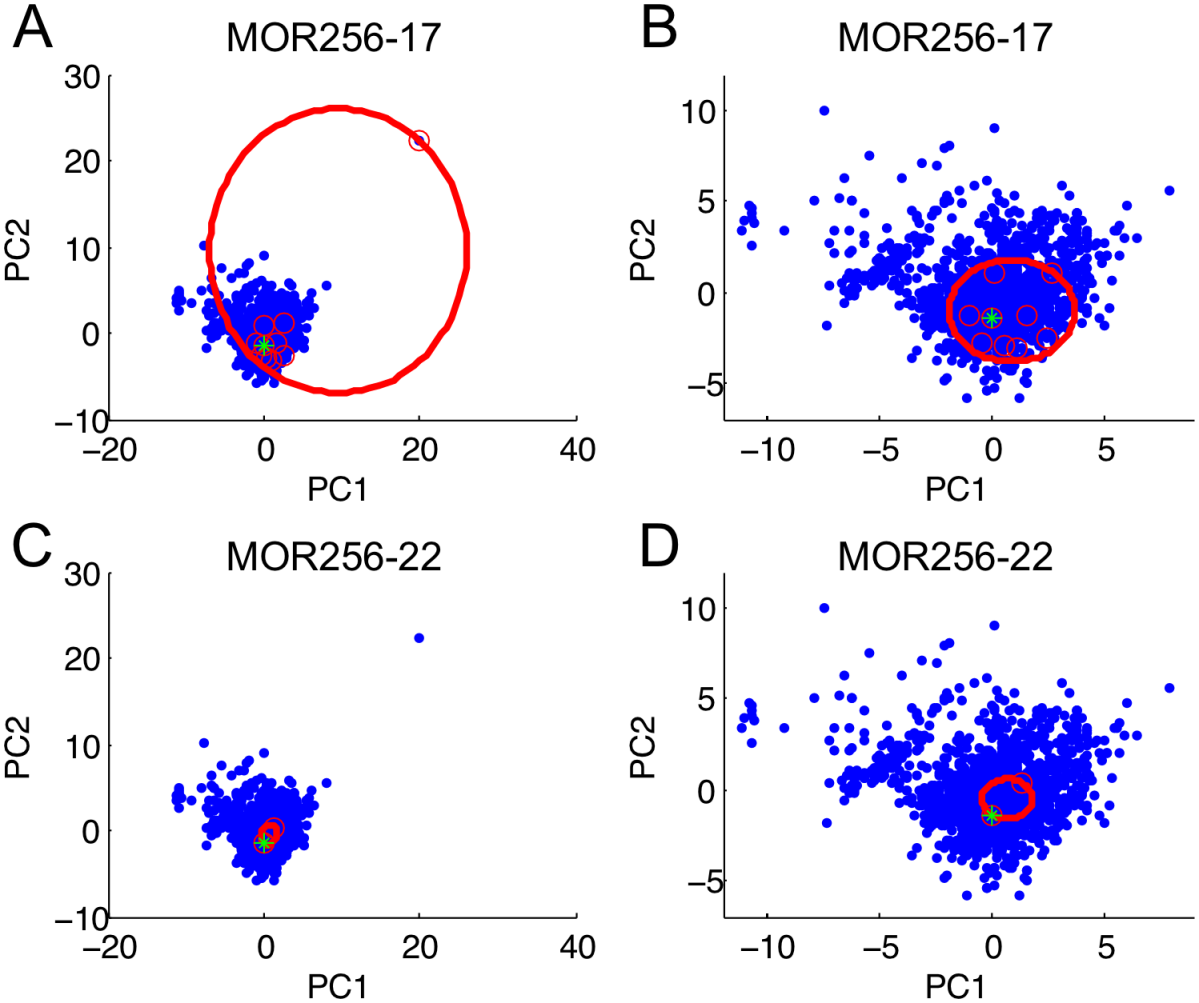
The molecular receptive range remains broad for MOR256-17 and narrow for MO256-22. (A,B) Active odorant ligands for MOR256-17 (red circles) are plotted within an estimated odor space. (A) The smallest hypersphere (indicated by the large red ellipse) that could encompass all odorants that activated the receptor had a radius of 38.6. (B) If iodoform was excluded, the value was 8.1. (C,D) Active odorant ligands for MOR256-22 (red circles) are plotted within an estimated odor space. The smallest hypersphere (indicated by the large red ellipse) that could encompass all odorants that activated the receptor was 3.4. Panels A and C show the entire odor space, including an extreme outlier (iodoform). Panels B and D show a close-up view of the region containing all of the odorants (excluding iodoform). The green star indicates *trans*-cinnamaldehyde.

To assess the tuning breadth for MOR256-17 and MOR256-22 when screened with our new odorant panel, we plotted the active odorants in a two-dimensional representation of an estimated odor space (Fig 5, S1 Table). The ten odorants that elicited MOR256-17 activity were well dispersed. The radius of a hypersphere encompassing the odorants that activated the receptor was 38.6 (Fig 5A). This value is exceptionally large, given that our estimated odor space has a radius of 45.6. However, the active odorants include the extreme outlier, iodoform. If iodoform is removed from the plot, the breadth of MOR256-17 receptor tuning is 8.1 (Fig 5B). This value remains larger than the value of 3.4 obtained for MOR256-22 (Fig 5C and 5D) and is a substantial portion of the radius encompassing all molecules in the space excluding iodoform (21.0). This tuning breadth value for MOR256-17 (with iodoform excluded) is also a substantial portion of the radius encompassing the odorants in our screening panel excluding iodoform (15.5). These results correlate well with Li et al., which obtained “tuning radii” of 16.4 for MOR256-17 and 4.8 for MOR256-22, using a distinct odorant panel [26].

## Discussion

Odor space, the array of compounds detected by olfactory systems, is vast and difficult to fully define [22, 25]. Practical considerations limit any screening panel that achieves broad coverage of odor space to a sparse coverage of the immense number of potential odorant structures. When screened with a broad but sparse odorant panel, ORs with different MRRs but similar tuning breadths, might appear to have different tuning breadths due to chance. That is, a large portion of the odorants that activate one of the ORs might happen to be in the panel by chance, while the panel might contain few of the odorants that activate the second OR, which might respond mainly to odorants not included in the panel. While an increasing number of mammalian ORs have been screened, few have been screened more than once. When ORs have been characterized in multiple studies, there has been substantial overlap among the odorant screening panels [21, 22, 26, 27]. Thus, the concern that variation in tuning breadth might be an artifact of our collective screening protocols cannot be easily dismissed.

Several members of the MOR256 subfamily appear to be broadly tuned [12, 21]. Li et al. subsequently examined the MRRs of three additional members of the MOR256 subfamily (MOR256-17, MOR256-8, and MOR256-22), using a diverse panel of 155 odorants [26]. By plotting the active odorants for each OR in an estimated odor space, Li et al. found MOR256-17 to be broadly tuned, responding to a highly diverse set of structures. In contrast, MOR256-22 was narrowly tuned, responding to just a few odorants that clustered in a small region of the estimated odor space. MOR256-8 appeared to have an intermediate tuning breadth. These results conformed well to the idea that mammalian ORs display a range of MRRs varying from narrow to broad. But because of the concerns discussed above, it remained possible that ORs such as MOR256-17 and MOR256-22 could in fact have similar tuning breaths, despite appearance to the contrary. Indeed, when MOR256-17 was screened with an odorant library of “~250” compounds [33], the 6 compounds that were identified as activators fell within a relatively small portion of an estimated odor space [26]. Unfortunately, the full composition of the screening panel was not provided, so it is difficult to judge how well that panel was distributed across odor space. Recently, the tuning breadth of MOR256-17 was examined in a mouse OSN context and was found to be broad [27]. The similarity of the set of active compounds identified in this study, to what was identified in the previous screening of MOR256-17 expressed in HEK293 cells [33] and *Xenopus* oocytes [26], supports the use of heterologous systems for OR screening. But, the significant overlap of the odorant screening panel used in the OSN study [27] with screening panels used in the earlier studies [26, 33], fails to allay concern about assessment of tuning breadth.

For these reasons, we decided to evaluate the tuning breadths of the apparently broadly tuned MOR256-17 and the apparently narrowly tuned MOR256-22 with an entirely new odorant screening panel that had a broad and well distributed coverage of an estimated odor space. We chose to examine these particular MORs because a previous detailed characterization of these receptors by our group [26] would then allow us to screen them under highly comparable experimental conditions. With the new screening panel, we asked whether the tuning breadths of MOR256-17 and MOR256-22 would retain their broad and narrow characteristics, respectively. We also screened MOR174-9 (mOR-EG), a well-characterized OR with a narrowly tuned MRR.

We found that these three MORs retained their previously assigned tuning breadths when tested with the new panel, supporting the idea that variation in tuning breadth among these three ORs is an intrinsic property of the receptors and not an artifact of screening panels. MOR256-17 responded to a wide variety of novel activators spread across a large portion of an estimated odor space. In contrast, MOR256-22 responded only to two closely related structures. The structural differences between the two activators of MOR265-22 suggest that the receptor favors aldehydes over alcohols. Alternatively, the additional dimethyl of α,α-dimethylbenzenepropanol (as compared to *trans*-cinnamaldehyde) could be a steric hindrance.

MOR174-9 did not respond to any of the odorants in our current (Table 1) or previous [26] screening panels. This receptor has previously been shown to respond to numerous compounds [28–30], but these compounds, benzene or cyclohexane rings (and polycyclic combinations) decorated with one or more oxygen containing moieties, have similar structures. Thus, MOR174-9 possesses a structurally restricted MRR and should be defined as narrowly tuned.

Human ORs have recently been screened with a panel of key food odorants (KFOs) [23, 34]. KFOs are thought to be the most relevant compounds underlying the aroma of foods and beverages [35]. When a human OR (OR1A1) was screened with a panel of 190 KFOs [23], the broadly tuned character of this OR was evident with applications in the micromolar range. However, a single KFO (3-methyl-2,4-nonanedione) was found to be exceptionally potent, activating this OR in the nanomolar range. When a single KFO (3-mercapto-2-methylpentan-1-ol) was used to screen 391 human ORs [34], a single OR (OR2M3) was found to respond to this ligand. Again, this responsiveness of a human OR to a KFO was found to be in the nanomolar range. When the entire KFO library, as well as additional compounds, were tested at higher concentrations, OR2M3 was found to be narrowly tuned, responding only to a few compounds closely related to the high potency KFO. These findings, for 2 human ORs, might generate concern that odorants other than the high potency KFOs are not relevant. That is, that detection of the high potency KFO ligands, perhaps only one KFO per OR, might be the only biologically relevant role for these ORs. A similar concern has been raised for insect ORs [36]. Such a strict interpretation would argue against a relevant role for combinatorial coding. However, it has been suggested that ORs might play multiple roles in representing odors [24], serving to detect particular KFOs at low concentration while also participating in combinatorial coding at higher concentrations. Thus, it is possible that the MOR256-17 and MOR256-22 receptors that we have examined here may also have multiple roles to play. The broad and narrow tuning breadths that we observe for these receptors may be important for combinatorial coding of complex odorant mixtures. But each of these MORs might also be highly selective for an as yet unidentified murine version of a KFO.

An interesting feature of the MRR of MOR256-17 is that while this receptor is broadly tuned, it is also selective. The great diversity of active structures is apparent when considering the various agonists of MOR256-17 identified in the current and previous work [26, 37]. MOR256-17 can be activated by simple linear compounds such as 2-heptanone and amyl methyl sulfide, but also responds to complex cyclic structures such as piperonal and 2,4,6-trinitrotoluene (TNT). At the same time, MOR256-17 is selective, responding to 6–7 carbon linear aldehydes, ketones and alcohols, but poorly or not at all to 4–5 or 8–11 carbon lengths. A recent study offers insight into how an OR might achieve both broad tuning and selectivity [38]. In this work, the effect of mutations on the odorant responsiveness of MOR256-3 (SR1) and MOR256-8 was examined. Critically, the tuning breadth of the narrowly tuned MOR256-8 could be substantially enlarged by mutating just one or two key residues. This broadening of the MRR was accompanied by an increase in receptor basal activity, suggesting a role in decreasing the receptor activation barrier. Thus, an OR with a moderately restrictive binding cavity and a low activation barrier might achieve both broad tuning and selectivity. Screening such ORs with an odorant panel chosen to adequately sample odor space provides a way to estimate the tuning breadth of OR MRRs.

## Materials and methods

### Materials

2,5-dihydro-2,4,5-trimethylthiazole was from Contech (Victoria, BC, Canada), pyridine and trimethylamine were from Alfa Aesar (Ward Hill, MA, USA). All other compounds and odorants were from Sigma-Aldrich (St. Louis, MO, USA). PubChem Compound Identification (CID) numbers for the odorants used in this study are listed in Table 1.

### Care and use of *Xenopus laevis* frogs

For this study, mature female *X*. *laevis* frogs were used as a source of oocytes. Frog care and use was carried out in accordance with the “Guidelines for Egg and Oocyte Harvesting in *Xenopus laevis*, Revised 07/14/10” from the Animal Research Advisory Committee of the Office of Animal Care and Use at the National Institutes of Health. The protocol was approved by the Institutional Animal Care and Use Committee of the University of Miami (Protocol Numbers: 13–056 and 13–149). 0.1% 3-aminobenzoic acid ethyl ester was used to anesthetize frogs. Sedation was assessed by loss of nasal flare and swallow reflexes. Oocytes were surgically removed and the incision was sutured. A subcutaneous injection of Baytril (0.05 mL of a 2.27% solution) was administered as an antibiotic and a subcutaneous injection of Meloxicam (0.1 mL of a 0.015% solution) was administered to the dorsal lymph sack to serve as an analgesic immediately following surgery. Before being returned to the holding tank, frogs recovered from surgery in a humid environment. Frogs had a rest period of at least 3 months between surgeries.

### Expression constructs

The nomenclature of Zhang and Firestein is used to refer to murine ORs (MORs) [10]. MOR256-17, MOR256-22, and MOR174-9 (mOR-EG) were cloned and inserted into the pCI vector (Promega) with an N-terminal extension consisting of the N-terminal 20 amino acid residues of human rhodopsin, as previously described [16, 26]. No other MORs were tested in this study. The human Gα_olf_ construct was purchased from the Missouri University of Science and Technology cDNA Resource Center. The human cystic fibrosis transmembrane regulator (CFTR) clone was kindly provided by Dr. Ian Dickerson (University of Rochester). Synthesis of cRNA encoding each protein was achieved with mMessage mMachine kits (Thermo Fisher Scientific).

### Preparation of oocytes and cRNA injection

To remove the follicle layer, oocytes were incubated for 2h with Collagenase B (Roche) at 22–25°C. Oocytes were injected with 46 nL of water containing cRNAs: 40 ng MOR, 10 ng Gα_olf_, 1.5 ng CFTR. A 2–4 day incubation period occurred post-injection at 18°C in Barth’s saline (in mM: 88 NaCl, 1 KCl, 2.4 NaHCO_3_, 0.3 CaNO_3_, 0.41 CaCl_2_, 0.82 MgSO_4_, 15 HEPES, pH 7.4 and 0.05 g/L tetracycline, 0.05 g/L ciprofloxacin, 0.1 g/L amikacin) prior to electrophysiological recording.

### Electrophysiology and data analysis

Electrophysiology and data analysis were performed as described previously [16, 26, 31, 39]. Two-electrode voltage clamp in an automated parallel electrophysiology system (OpusExpress 6000A, Molecular Devices) was used to measure odorant induced Cl^-^ currents, resulting from cAMP-mediated activation of the co-expressed CFTR reporter channel [40]. Micropipettes with resistances of 0.2–2.0 MΩ were filled with 3 M KCl. The holding potential was -70 mV. *OpusXpress* 1.1 software (Molecular Devices) was used to capture and store current responses, filtered (4-pole, Bessel, low pass) at 20 Hz (-3 db) and sampled at 100 Hz. *Clampfit 9*.*1* software (Molecular Devices) was used to perform initial analysis. Oocytes were perfused with ND96 (in mM: 96 NaCl, 2 KCl, 1 CaCl_2_, 1 MgCl_2_, 5 HEPES, pH 7.4). Odorants were stored under argon gas and high concentration (0.5 M) stock solutions of each odorant were prepared in dimethylsulfoxide or ethanol. Each odorant, diluted in ND96 to 30 μM, was applied for 15 s, followed by a 20-min wash with ND96. A wide variety of chemical structures can directly activate the CFTR [41], raising the possibility of false positive responses in our screening system. However, none of the odorants in our screening panel elicited a current response when applied to oocytes expressing MOR174-9 (a structurally restricted MRR), Gα_olf_ and CFTR; ensuring that the odorants in the panel were not acting directly on the CFTR at our screening concentration of 30 μM. Also, when applied to sham (water) injected oocytes, none of the odorants in our screening panel elicited current responses.

### Selection of odorant screening panel and estimation of odor space

To obtain a panel of structurally diverse odorants, we employed a multidimensional odor metric that is based on 32 physiochemical descriptors [25]. We obtained molecular structure files from PubChem (http://pubchem.ncbi.nlm.nih.gov/) and used Dragon software (Talete) to compute physiochemical descriptors. Principal component analysis was used to depict the molecules in two dimensions for Figs 1 and 5. We plotted odor space using 1595 molecules common to olfactory studies [25, 42]. For this study, we selected a panel of 60 compounds to represent the various regions of our estimate of odor space using a previously published set of odorant selection groups [25] to choose compounds that adequately sampled various regions of the estimated odor space. We estimated the breadth of receptor tuning as the radius of the smallest hypersphere that could encompass all molecules that activated a receptor.

### Screening protocol

We initially divided our new odorant panel into 6 mixtures, each containing 9–10 odorants at 30 μM (Table 1). The response to each mixture was compared to the response to 30 μM of a known odorant activator of that receptor (normalizer): 2-heptanone for MOR256-17, *trans*-cinnamaldehyde for MOR256-22, or eugenol for MOR174-9. Compounds were considered active if they reproducibly evoked responses greater than 1% of the normalizer. Two mixtures were tested on each oocyte. To account for potential receptor desensitization following a robust response, mixtures were also tested in reverse order (for example, mixture 1 followed by mixture 2 for some trials, mixture 2 followed by mixture 1 for others). Because response amplitudes are variable within and between oocytes batches, odor evoked responses are always presented as a percentage of the normalizer response. As a positive control, a compound known to activate both MOR256-17 and MOR256-22, *trans*-cinnamaldehyde [26], was included in mixture 4. A delay in obtaining cyclodecanone resulted in this compound being tested individually. Additionally, because of the apparent reactivity of iodoform (see Results), it was removed from its mixture early in our studies and was subsequently tested individually. 1,3,4,6,7,8-hexahydro-4,6,6,7,8,8-hexamethylcyclopenta[g]-2-benzopyran (included in mixture 3) was obtained in solution with diethyl phthalate (50%). This made diethyl phthalate the unintentional 61^st^ chemical in the panel. We did not observe any receptor responses for either compound.

## Supporting information

S1 Table Data underlying Figs 1 and 5. (XLSX)

S2 Table Data underlying Fig 2. (XLSX)

S3 Table Data underlying Fig 3. (XLSX)

S4 Table Data underlying Fig 4. (XLSX)
